# Effect of the COVID-19 Pandemic on Respiratory Diseases and Their Economic Impacts

**DOI:** 10.3390/pathogens13060491

**Published:** 2024-06-08

**Authors:** Ananya Sivaraman Jayaraman, Ishita Darekar, Nidhi Vijayprakash Dadhich, Lakshmi Sai Manasvi Tadepalli, Yao Gongwang, Sunil Singh, Edem Gavor

**Affiliations:** 1 Global Indian International School, 27 Punggol Field Walk, Singapore 828649, Singapore; sivaramanananya@gmail.com (A.S.J.); ishita.s.darekar@gmail.com (I.D.); nidhi.v.dadhich@gmail.com (N.V.D.); tadepallimanasvi@gmail.com (L.S.M.T.); 2 Department of Biological Sciences, National University of Singapore, Singapore 117543, Singapore; e0950135@u.nus.edu (Y.G.); a0123798@u.nus.edu (S.S.)

**Keywords:** COVID-19, respiratory diseases, influenza, tuberculosis, asthma, economic impact

## Abstract

COVID-19 is an airborne respiratory disease that mainly affects the lungs. To date, COVID-19 has infected 580 million people with a mortality of approximately 7 million people worldwide. The emergence of COVID-19 has also affected the infectivity, diagnosis, and disease outcomes of existing diseases such as influenza, TB, and asthma in human populations. These are airborne respiratory diseases with symptoms and mode of transmission similar to those of COVID-19. It was speculated that the protracted nature of the COVID-19 pandemic coupled with vaccination could impact other respiratory diseases and mortality. In this study, we analyzed the impact of COVID-19 on flu, tuberculosis (TB), and asthma. Our analyses suggest that COVID-19 has a potential impact on the mortality of flu, TB, and asthma. These impacts vary across before the COVID-19 era, during the peak period of the pandemic, and after vaccinations/preventive measures were implemented, as well as across different regions of the world. Overall, the spread of flu generally reduced during the pandemic, resulting in a reduced expenditure on flu-related hospitalizations, although there were sporadic spikes at setting times. In contrast, TB deaths generally increased perhaps due to the disruption in access to TB services and reduction in resources. Asthma deaths, on the other hand, only marginally varied. Collectively, the emergence of COVID-19 added extra cost to the overall expenditure on some respiratory infectious diseases, while the cost for other infectious diseases was either reduced or somewhat unaffected.

## 1. Introduction

The coronavirus disease (COVID-19), which was caused by the severe acute respiratory syndrome coronavirus-2 (SARS-CoV-2), is an airborne respiratory disease that mostly affects the lungs and leads to several complications that may include pneumonia, acute respiratory distress syndrome, multi-organ failure, septic shock, and death [1,2,3]. The outbreak started in 2019, and by March 2020, the World Health Organization (WHO) declared it a pandemic [4]. The most common symptoms of COVID-19 are headache, fever, cough, tiredness, and loss of taste or smell [5]. SARS-CoV-2 can spread from an infected person’s mouth or nose in air droplets [6]. People with existing medical problems like cardiovascular disease, diabetes, chronic respiratory disease (such as asthma), and cancer and immunocompromised patients are most likely to develop serious illness [7]. As of 25 October 2023, SARS-CoV-2 has infected over 700 million people, killing almost 7 million people worldwide according to the WHO [8]. The preventative measures for COVID-19 include vaccination, social distancing, quarantining, hand washing, and the use of sanitizers and face masks to minimize the risk of transmission [9].

The emergence of COVID-19 can affect the infection, diagnosis, disease severity, and disease outcomes of existing pathogens or diseases in different ways. (1) During the pandemic, most public health resources were dedicated to curbing, preventing, and ultimately eradicating COVID-19. As such, other existing diseases may be neglected. (2) Because of the similarity between COVID-19 symptoms and other diseases symptoms, diagnosing and differentiating these symptoms can be problematic. (3) COVID-19 compromises the immune system, allowing other pathogens to easily infect and cause disease [10]. (4) Furthermore, adjustment in overall lifestyle, including better and more stringent hygiene practices, social distancing, reduced air travel, and the use of personal protective equipment such as masks could curb the spread of other respiratory infections. (5) Lastly, intensive vaccination programs against COVID-19 may positively affect the prognosis of other similar pathogens.

Because COVID-19 affects the respiratory system [11,12,13], it could exacerbate the conditions of unvaccinated people already living with other respiratory problems. Overall, COVID-19 could either positively or negatively affect other disease cases and mortality in human populations. Before the COVID-19 pandemic, other respiratory-associated diseases caused by viruses such as the influenza or flu viruses, bacteria (such as tuberculosis (TB)), or non-pathogenic diseases such as asthma (due to pollen, dust mites, mold spores, etc.) were already a major public health concern. The present study considered these diseases in detail. Tuberculosis (TB) is caused by the bacterium *Mycobacterium tuberculosis*, while influenza viruses are members of the family Orthomyxoviridae responsible for causing influenza or the flu [14]. Like COVID-19, TB and influenza are spread through inhaling tiny droplets from the coughs or sneezes of an infected person [15], primarily affecting the lungs. Furthermore, TB, influenza, and COVID-19 share symptoms such as cough, pain in the chest, fatigue or weakness, loss of appetite, chills, fever, and night sweats [16]. According to the CDC, 1.7 billion people were infected with TB in 2018, representing 23% of the world’s population [17].

TB is the leading infectious disease killer in the world, claiming 1.5 million lives each year [17]. In 2020, this number decreased to 10 million global TB infections, although mortality was still high [16]. The WHO estimates that seasonal influenza may result in 290,000–650,000 deaths each year due to respiratory diseases alone [18]. Like TB, influenza, and COVID-19, asthma is a disease that primarily affects the lungs [19]. However, unlike these three diseases that are caused by pathogens, asthma development and complications have been linked to genetic, environmental, and occupational factors [19]. Patients exhibit wheezing, breathlessness, chest tightness, and coughing. Asthma affected an estimated 262 million people in 2019 and caused 455,000 deaths. In 2020, deaths due to asthma in the United States skyrocketed for the first time in 20 years, killing over 4145 more people [20].

Because these three diseases affect the lungs and upper respiratory airways with similar disease symptoms to those of COVID-19, it is speculated that the protracted nature of the COVID-19 pandemic coupled with vaccination programs could impact these three disease scenarios and mortality. While some studies have attempted to look at the impact of COVID-19 on these diseases [15,21,22], a comprehensive analysis and comparison of the disease’s burdens before the COVID-19 pandemic, during the COVID-19 pandemic (without vaccination programs), and during the COVID-19 pandemic (with vaccination programs) across different countries are still lacking.

Here, we sought to analyze the positive and negative impacts of COVID-19 on influenza (or flu), tuberculosis (or TB), and asthma as a representative of viral, bacterial and non-pathogenic respiratory illnesses. We curated data by leveraging the disease morbidity and mortality information of reputable public health databases such as the WHO, the Centers for Disease Control and Prevention (CDC), research article publications, and news reports. Our aim was to analyze the impact of the COVID-19 pandemic on the mortality rate of the flu, TB, and asthma before the COVID-19 era, during the peak period of the pandemic, and after vaccinations and other preventive measures were implemented. We also aim to understand how the change in the death rates of these respiratory diseases might have affected the economy in terms of patient care, and ultimately, what the prognosis might be moving forward.

## 2. Methods

To understand the impact of COVID-19 on the flu, TB, and asthma, we searched the literature and databases for the mortality of these three diseases across seven representative countries: North America (United States of America and Canada), Europe (United Kingdom and Germany), Asia (Japan and Republic of Korea), and Australia. We looked at the mortality rates from the year 2016 to 2021. We divided our analysis into three categories: (1) prior to 2019 was regarded as the pre-COVID-19 era; (2) late 2019 to 2020 was the era of the COVID-19 pandemic with no therapeutics or vaccines, and symptom-based treatment was employed; and (3) the COVID-19 era from 2021 to now, where vaccines [2], therapeutics, monoclonal antibodies [2], and antiviral drugs [3,23] are available.

Initially the literature survey was conducted using PubMed (https://pubmed.ncbi.nlm.nih.gov/) and Google to identify three distinct respiratory diseases, which were affected by COVID-19. Further keyword searches were performed to identify the impact of COVID-19 on viral infection (flu), bacterial infection (TB), and infection due to other factors (asthma) as representative diseases from different pathogenic and non-pathogenic origins. Subsequently, systematic searches were conducted with the databases from the World Health Organization (WHO), the Centers for Disease Control and Prevention (CDC) (https://www.cdc.gov/), the Global Health Expenditure Database (https://apps.who.int/nha/database/) and on other reliable sources, as well as the literature for extracting the key details of flu, TB, and asthma.

Since the number of infections from various sources vary, we report only the mortality numbers in the tables. These values were mostly obtained from the WHO mortality database (https://www.who.int/data/data-collection-tools/who-mortality-database), which is mostly up to date up to 2019, and for some of the countries, it is up to date up to 2020 at this moment. The missing mortality numbers for some of these countries for the years 2020 and 2021/2022 were collected from other reliable sources that we have indicated in the footnotes or appropriate sources cited in the tables as references. It must be noted that some of the details on these three diseases for the recent years (2022–2023) still need to be updated in the public domains and the literature, and thus, the data are not thorough or complete at this moment and need to be approached cautiously.

## 3. Results and Discussion

The current study was predicated on the hypothesis that the COVID-19 pandemic (being a respiratory infection) may have impacted other respiratory infections and conditions in human populations. Our main objectives for this study were to find out if the COVID-19 pandemic affected the number of deaths resulting from respiratory diseases such as the flu, TB, and asthma and to point out differences before the pandemic, during the height of the pandemic, and when vaccines and therapeutics became available. In this study, we sought to provide informed suggestions on how future pandemics might be responded to with regard to other diseases.

### 3.1. Impact of COVID-19 on Influenza (Commonly Known as the Flu)

**Figure 1:**
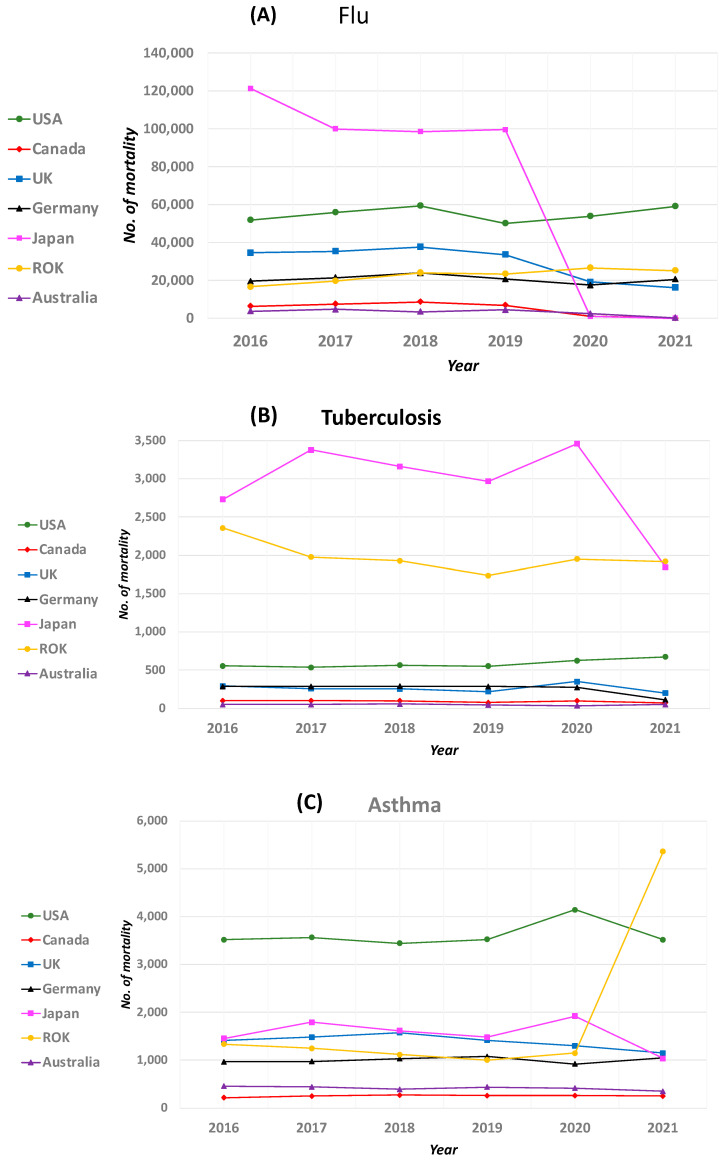
(**A**) shows the plot between the number of mortality due to the flu and year for the selected seven countries as representative from various continents. There were no significant differences in the mortality numbers observed during the pre-COVID era (i.e., 2019 and before) compared to during the pandemic (i.e., early 2020 and onward). For the specific details and numbers, please refer to Table 1. Figure 1A was created with Microsoft excel, version 2019 (**B**) shows the plot between the number of mortality due to TB and year for the selected seven countries as representative from various continents. There were no significant differences in the mortality numbers observed during the pre-COVID era (i.e., 2019 and before) compared to during the pandemic (i.e., early 2020 and onward). For the specific details and numbers, please refer to Table 2. Figure 1B was created with Microsoft excel, version 2019. (**C**) shows the plot between the number of mortality due to asthma and year for the selected seven countries as representative from various continents. There were no significant differences in the mortality numbers observed during the pre-COVID era (i.e., 2019 and before) compared to during the pandemic (i.e., early 2020 and onward). For the specific details and numbers, please refer to Table 1. Figure 1C was created with Microsoft excel, version 2019.

Seasonal influenza outbreaks are usually reported in the months of October through to May or June every year [15]. One of the positive aspects of the COVID-19 pandemic was a drop-in influenza infection and mortality [15]. From our analysis, we observed that prior to COVID-19 (i.e., before March 2019), influenza-related mortality was significantly higher across all of the seven studied countries (Table 1, Figure 1A). 

A decline in influenza mortality was observed during the peak of COVID-19 (late 2019 to 2020), and by 2021, the overall mortality rate of influenza was significantly unexpectedly low in Canada, Japan, and Australia. In Canada, the average 7500 deaths/year (2016 to early 2019) was reduced drastically to 1183 in (5 January 2020–3 October 2020) according to Canada’s Flu Watch report [22]. During the 2020–2021 Canadian influenza season (23 August 2020–28 August 2021), no community circulation of influenza or influenza-associated death was reported, and the influenza infection was historically low compared with the previous six seasons [22]. Unexpectedly, the number of influenza deaths started rising again in Canada from 15 May 2022 to now (94 deaths), which is a rare situation, as influenza cases are expected to be low during the spring season [26,43]. This has been attributed to the easing of COVID-19 restrictions. According to the Public Health Agency of Canada’s (PHAC) most recent Flu Watch report, the number of flu cases remains above the epidemic threshold [26].

In Germany, while an average of over 20,000 deaths due to flu were recorded from 2016 to 2019, the country recorded a decline in mortality (17,593 deaths) in 2020. The same downward trend was observed for Japan and Australia, where the 2016–2019 influenza deaths were, on average, 100,000 and 4000 respectively, but decreased to only 965 (in 2020) and 2548 (in 2020), respectively, and only 295 deaths were recorded in Australia in 2021 (Table 1).

In Northern Hemisphere countries like the USA, pre-COVID-19 influenza mortality was above 50,000/year. Interestingly, while this number was predicted to decrease to 4700–1400 by 2021, the number of flu deaths was equivalent to one of the highest death rates since 2016, with a record number of over 59,000 deaths (Table 1). Despite this record high number of deaths, the number of flu infections detected during the period of 2020–2021 was relatively low, as expected from the expected number of predicted deaths. For example, for the period between 28 September 2020 and 22 May 2021, out of the 818,939 respiratory specimens tested in the USA, only 1675 (0.2%) were positive for influenza virus compared to the pre-COVID-19 era; there were two consecutive flu seasons with peak infection rates between 26.2% and 30.3% [44]. According to the CDC, this decline accounts for the lowest rate recorded since 2005 [44].

Thus, the decline in the infection rate was expected to contribute to fewer influenza illnesses, hospitalizations, and deaths compared to previous seasons. Similarly, South Korea recorded an increase in the number of flu deaths in 2020 (26,650 deaths) and 2021 (25,176 deaths) compared to the previous 2016–2019 average of about 21,000 deaths/year (Table 1).

The rise in the flu mortality rate might also be due to the increase in accessibility of the flu shot or vaccinations, which resulted in over 36 deaths as of October 2020, thwarting the vaccination effort [45].

Notably, the influenza circulation and death in the entire world was at a historical low in the Northern (Canada, US, and Europe) and the Southern (Australia) Hemispheres, as well as in other countries during the COVID-19 era compared with the pre-COVID-19 era (Table 1). The worldwide overall mortality due to influenza-associated deaths from respiratory causes alone was 290,000–650,000, as reported in a 2017 study [46], while a 2019 study estimated 99,000–200,000 deaths [46]. During the COVID-19 period of the years 2020–2021, however, the global influenza disease burden was at an all-time low with a reduced mortality rate, although the flu public health reports for 2022 is not available for any of the countries studied (Table 1). These numbers will be necessary to clearly understand the trajectory of the effect of COVID-19 after rigorous vaccination programs are reduced.

One explanation for the observed decline in flu mortality could be that since hospitals and government organizations were overwhelmed during COVID-19, there was less reporting of influenza-related deaths. However, flu activity was unusually low throughout the 2020–2021 flu season globally (with the exception of the United States and South Korea), despite high levels of testing, according to the CDC [44]. In Canada, testing through the 2020–2021 season was roughly twice the historical average while the reported percent positivity ranged from 0.0% to 0.1% for this season compared to a historical average range of 0.8% to 25.1% [22]. 

Globally, the implementation of COVID-19 public health measures to mitigate the spread of the coronavirus might be one of the reasons for the drastic reductions in influenza infections and mortality [47]. However, this situation also warrants increased attention as the decreased spread of influenza might have lowered the level of immunity among the population, and this can lead to higher infections if the influenza re-circulates [22].

Moreover, as the COVID-19 vaccination drive is almost completed in most of the countries, leading to the ease of public health measures, an influenza spread similar to that in the pre-COVID-19 era may result in large influenza epidemics, as observed in the United States and South Korea. However, mass flu vaccination and the introduction of new, more effective vaccines against mutant flu viruses [48] might significantly contribute to reducing the spread of the influenza infection and death during the pandemic period and post-pandemic era.

### 3.2. Impact of COVID-19 on Tuberculosis (TB)

In the early stage of the COVID-19 pandemic, theoretical models predicted hundreds of thousands of additional TB cases annually due to the disruption of health care services [49]. In particular, the TB mortality was predicted to increase by 5–15% over the next 5 years worldwide, especially due to additional complications from COVID-19 [49].

Similar to our analysis on flu, we collected TB mortality data for the seven countries of interest (Table 2; Figure 1B). 

The currently available evidence suggests that there has been some level of disruption to TB health services. Despite this, from our analysis, we observed a discrepancy in TB mortality from one country to another. For example, health care resources have been diverted to COVID-19 treatments, perhaps leading to an increased number of TB cases in some countries such as the USA and Canada. However, in other instances, TB mortality decreased in countries such as the UK and Germany. In the cases of the Republic of Korea and Australia, TB mortality has not varied significantly.

From 2016 to 2019, the TB related mortalities in these countries were mostly constant with small variations, while in 2020, TB mortality generally increased. For instance, the average TB mortality prior to 2019 in the US was around 550/year; however, in 2020, the mortality increased to 625 (Table 2). In 2021, effective vaccines against COVID-19 became available. Vaccines were expected to reduce the rate of SARS-CoV-2 co-infection with TB and ultimately reduce the level of complications that COVID-19 had on TB patients. Among the seven countries, Japan experienced a significant decrease in TB deaths. However, data on TB-related deaths were not available for 2022–2023 in all the countries at the time of writing this manuscript. Hence, it is unclear what the current trend is for TB-related deaths.

It is not clear how COVID-19 and the measures taken to control the SARS-CoV-2 virus spread may have influenced or affected the TB transmission. At best, two scenarios can be used to explain the reason for increased TB cases in some countries and declines in other countries. Despite mask wearing and social distancing, which might decrease the TB transmission, due to lockdowns, the mobility of TB patients was restricted, and they were forced to stay at home. Thus, there were increased household spread; increased delay in testing and treatment; decreases in TB vaccination (BCG—Bacillus Calmette–Guérin vaccine), coverage, and drive; decreases in TB testing and treatment enrollment; increases in treatment interruptions; and, therefore, increased vulnerability to TB [49]. These are some of the potential impacts of COVID-19 on the TB health services and treatments and might be the reasons for the increased numbers of TB mortality in the USA and Canada. A similar trend of increases in the TB mortality was also observed in high-risk TB burden countries like India and African countries, although these countries are not the focus of our study. In India, for example, prior to the COVID-19 era, there were 449,000 TB deaths reported in 2018, while in 2020 and 2021, this number became 500,000 [50] and 504,000 [51], respectively.

SARS-CoV-2 co-infection with TB was a major concern for the WHO, posing the threat of exacerbating TB symptoms and increasing deaths. When COVID-19 vaccines became available, this co-morbidity scenario was expected to improve. However, in these countries, TB vulnerability is likely to continue to increase despite the COVID-19 vaccines and treatments that are available, due to the continuous spread of different mutants of the SARS-CoV-2 virus, which evades immunity [52,53]. For example, the total number of TB patients recorded in India dropped by 25% in 2020, and then increased to 19% in 2021 [51]. Most of the TB high-risk countries are low- or mid-income countries, and poverty remains a challenge. Moreover, the impact of multi-drug resistance in TB treatment cannot be ignored [51].

### 3.3. Impact of COVID-19 on Asthma

Next, as a part of our analysis on the impact of COVID-19 on other respiratory infectious diseases, we sought to analyze mortality due to asthma during the COVID-19 pandemic. The effects of COVID-19 on people with asthma are complex due to various factors [54]. Intuitively, a disease such as COVID-19 that attacks the lungs or respiratory system should put asthma sufferers at much greater risk. Similar to our analyses of TB and flu infections, the mortality due to asthma in the selected seven countries prior to 2019 was constant, while during the pandemic, the mortality varied marginally (Table 3; Figure 1C). 

For example, there were marginal increases in asthma deaths in the USA and Canada in 2020. In the other countries, it marginally decreased. The total mortality due to asthma in the world prior to COVID-19 in 2019 was 455,000 [55]. Apart from South Korea, which recorded a significantly high mortality rate of asthma in 2021 (from an average of approximately 1300 since 2016 to 5357 in 2021), the rest of the countries maintained a fairly stable number of deaths due to asthma. Overall, the mortality due to asthma during COVID-19 has not varied significantly.

**Table 3:** Numbers of deaths recorded from asthma.

III		Numbers of Deaths (2016–2021) (Due to Airborne Allergens like Pollen, Dust Mites, Mold Spores, Pet Dander, or Particles of Cockroach Waste)							
S. No	Country	2016	2017	2018	2019	2020	2021	Overall Changes Due to COVID-19	References
1	USA	3518	3564	3441	3524	4145	3517	Decreased ↓	[20,24,56]
2	Canada	214	249	269	260	262	250	No obvious change	[24,57,58]
3	UK	1409	1482	1579	1415	1302	1146	Decreased ↓	[24,59,60]
4	Germany	9671	9711	10,341	1077	924	1049	Increased ↑	[24,61,62]
5	Japan	1454	1794	1617	1481	1922	1038	Decreased ↓	[24,63,64]
6	Republic of Korea	1337	1250	1117	1004	1150	5357	Increased ↑	[24,65]
7	Australia	459	444	392	436	417	351	Marginally decreased ↓	[24,66]

Red: significant increase; green: significant decrease; yellow and orange: marginal differences.

Thus, the expected threat of COVID-19 to asthma patients did not turn out to be a problem due to various reasons. Many patients take preventive medicines for asthma such as inhaled corticosteroids, a type of steroid drug that reduces the amount of inflammation in the lungs [67]. Interestingly, dexamethasone, another steroid drug being used as a treatment for COVID-19, also reduces inflammation in the lungs. Incidentally, asthmatics who use these steroids might be inadvertently reducing the risk of severe COVID-19 because they are sort of “pre-treated” against the disease. Indeed, asthmatics who use preventers are thought to be “anti-SARS-CoV-2”, and somewhat immune to COVID-19 [68]. Furthermore, a study from Australia demonstrated that patients with asthma have decreased expression of the SARS-CoV-2 entry receptor, ACE2 [68]. A lower ACE2 expression means less gateways for the virus to enter our cells and cause COVID-19. Lastly, asthma is a chronic condition that flares up because of triggers such as pollens, chemicals, dust mites, pets, mold, smoke, or viruses. COVID-19 restrictions, including lockdowns, the wearing of masks, and social distancing, may have protected asthmatics from these triggers.

### 3.4. Economic Impact of COVID-19

The WHO’s Global Health Expenditure Database (https://apps.who.int/nha/database) indicates that the global spending on health was USD 8.5 trillion in 2019, or 9.8% of the global GDP. However, it was unequally distributed, with high-income countries accounting for approximately 80% of the health spending. According to the World Bank income group, all the seven countries in Table 1, Table 2 and Table 3 are high-income countries. An early estimate of the health spending during the COVID-19 pandemic in the high-income economies indicated that health spending increased substantially in 2020, more than that in previous years, due to COVID-19-related treatment costs followed by testing/tracing and medical goods.

During the COVID-19 pandemic, the seasonal flu infection was unexpectedly low, and this has positively impacted the economy. The total economic burden of seasonal influenza incorporates both direct (e.g., hospitalization) and indirect costs, such as those due to absence from work or premature death. However, studies were not always clear about the exact components included in the assessment of total costs. For instance, in the pre-COVID-19 era, the annual flu costed the U.S. approximately USD 10.4 billion in direct costs for hospitalizations and outpatient visits for adults. In 2017, a study from Canada reported that each case of influenza hospitalization cost the health care system CAD 14,000–CAD 20,000. With an estimated 12,000 influenza hospitalizations annually, the cost to the Canadian economy is CAD 168–CAD 240 million [67]. During the COVID-19 pandemic, this expense was significantly reduced (https://www.cdc.gov/flu/).

On the other hand, the COVID-19 pandemic has reversed years of global progress in tackling TB, and for the first time in a decade, TB deaths have increased (WHO report 2021). Compared to 2019, in 2020, more people died from TB with fewer people diagnosed and treated for TB. The key reason may lie in the disruption of access to TB services and a reduction in resources. In many countries, the resources have been reallocated from TB to COVID-19, which limit the essential health care services for TB diagnosis and treatment. This needs to be immediately addressed with additional resources and investment to prevent and treat the TB infections, as the impact of the COVID-19 pandemic on TB has been particularly severe. For example, approximately, 1.5 million people died from TB in 2020 (including 214,000 among HIV-positive people). The WHO’s modelling projections suggest that the number of people developing TB and dying from the disease could be much higher in 2022. The number of people newly diagnosed with TB fell from 2019 to 2020.

The WHO estimates that approximately 4.1 million people currently suffer from TB but have not been diagnosed with the disease. Taken together, all these factors indicate that TB has an adverse impact on the economy. Similar to TB, asthma also has an aggravated negative impact on the economy. Combining missed workdays, medical expenses, and deaths, the American Thoracic Society estimates that the U.S. alone spends approximately USD 80 billion on asthma-related costs each year [69].

## 4. Conclusions

**Figure 2:**
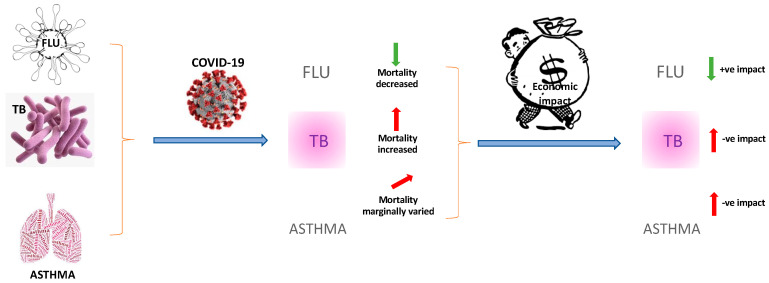
Schematic of effects of COVID-19 on other respiratory diseases such as the flu (viral infection), tuberculosis (TB—bacterial infection), and asthma (infection due to other factors) and their economic impacts. Flu mortality decreased due to COVID-19; however, the mortality rates of TB and asthma increased and marginally varied, respectively. Consequently, the economic impact of the flu due to COVID-19 decreased (i.e., less expenses for the flu), while TB and asthma demand more resources and, thus, require more financial supports. Figure 2 was created with Microsoft Power Point, version 2019.

The COVID-19 pandemic has impacted the mortality rates of other respiratory diseases both positively and negatively, although it is unclear if there are other contributing co-morbidities (Figure 2). 

Our analysis of the databases, combined with the literature, suggests that the COVID-19 pandemic led to a decrease in flu-related mortalities between the periods of 2020 and 2021 in most countries with the exception of the United States and South Korea, which observed higher numbers of deaths in 2021, contrary to previous predictions. However, this might not be permanent, and mortality may return to pre-pandemic levels or even higher as COVID-19 restrictions are gradually being removed globally, as again exemplified by the high death rates recorded in 2021 in the United States and South Korea. People might become more complacent in their day-to-day activities, and this could lead to a spike in other respiratory infectious diseases and death. On the other hand, the impacts of COVID-19 on TB and asthma seem to vary from country to country. In some countries, such as the United States and Canada, the TB mortality rate has increased, while in the rest of the countries except Japan and Germany, where TB cases have reduced significantly, the mortality rates have remained fairly constant over the pre-pandemic, pandemic and the post-pandemic eras. The reason for these disparities is unclear at the moment. However, in countries where cases have increased, one reason could be due to the diversion of resources from TB to COVID-19.

Overall, across the seven countries, during COVID-19, the rate of mortality due to influenza decreased, the rate of mortality due to TB increased, and the rate of mortality due to asthma does not reveal a significant impact of COVID-19 in these countries. This may be because, unlike TB, which is a bacterial infection, and asthma, which is non-pathogenic, influenza is a viral infection with a specific seasonal outbreak. Moreover, influenza virus has a different life cycle compared to that of TB.

However, the current analysis is inexhaustive, as we could not find data for the years 2022–2023. It would be interesting to further analyze the recent trajectory of these three and other respiratory infections from 2022 to long after the implementation of COVID-19 vaccination programs.

Overall, the emergence of the COVID-19 pandemic certainly added an extra cost to the overall expenditure on respiratory infectious diseases globally. Moving forward, as COVID-19 gradually rescinds, causing countries around the world to lessen restrictions, it is advised that extreme care and caution is taken to avoid unexpected spikes in under-studied and other respiratory infectious diseases.

## 5. Limitations of the Study

In our study, we focused on diverse sources of information to curate the numbers of deaths recorded for the flu, TB, and asthma during the COVID-19 pandemic, considering specific countries that possibly represent the scenario in different continents. We focused on forecasts from reputable organizations, news reports, and research articles. It is important to note that some details about these three diseases from recent years (2022–2023) have yet to be updated in public records and the literature. As a result, the information is currently neither comprehensive nor complete and should be approached with caution. A second limitation is that we did not capture studies from crucial regions of the world, such as the African continent and other highly populated regions like India and China, where large numbers of flu, TB, and asthma cases are recorded each year. Our rationale, however, was to focus on regions with relatively well-updated forecasts on these diseases. Perhaps future studies focusing on other parts of the world would help provide a more accurate and global picture of the effects of COVID-19 on these three diseases or consider additional respiratory diseases. Also, examining the numbers of deaths and comparing them across countries and categories may not fully reflect the real changes, as these studies may have examined different populations at different times. However, we are confident that the current studies provide a general idea of how COVID-19 may have affected the impact of these three studied respiratory diseases. A study that focuses exclusively on the effects of COVID-19 on different populations at specific periods could be an important study in the future. Finally, because there have not been studies that looked at the direct correlates of the economic impact of the COVID-19 pandemic on the flu, TB, or asthma, the economic analysis reported here involves extrapolations. 

## Figures and Tables

**Table 1:** Numbers of deaths recorded from flu infections.

I		Numbers of Deaths (2016–2021) Flu Viral Lower Respiratory Infections (Viral Infection)							
S. No	Country	2016	2017	2018	2019	2020	2021	Overall Changes Due to COVID-19	References
1	USA	51,850	56,027	59,490	50,133	53,918	59,215	Increased ↑	[24,25]
2	Canada	6337	7499	8657	6969	1183	94	Decreased ↓	[24,26]
3	UK	34,672	35,399	37,646	33,577	19,353	16,237	Decreased ↓	[24,27]
4	Germany	19,592	21,416	23,933	20,684	17,593	20,656	Increased ↑	[24,28]
5	Japan	121,381	100,019	98,539	99,664	965	22	Decreased ↓	[24,29]
6	Republic of Korea	16,736	19,704	24,063	23,467	26,650	25,176	Decreased ↓	[24,30]
7	Australia	3766	4759	3479	4510	2548	295	Decreased ↓	[24,31]

Red: significant increase; green: significant decrease; yellow and orange: marginal difference.

**Table 2:** Number of deaths recorded from tuberculosis infections.

II		Numbers of Deaths (2016–2021) (Bacterial Infection)							
S. No	Country	2016	2017	2018	2019	2020	2021	Overall Changes Due to COVID-19	References
1	USA	554	534	564	551	625	673	Increased ↑	[24,32]
2	Canada	100	101	99	77	97	73	Decreased ↓	[24,33,34]
3	UK	292	256	255	220	351	201	Decreased ↓	[24,35,36]
4	Germany	285	287	291	286	275	112	Decreased ↓	[24,37]
5	Japan	2731	3377	3160	2965	3459	1844	Increased ↑	[24,38,39]
6	Republic of Korea	2355	1977	1929	1738	1950	1917	Decreased ↓	[24,40,41]
7	Australia	49	49	55	45	33	51	Increased ↑	[24,42]

Red: significant increase; green: significant decrease; yellow and orange: marginal difference.
